# Marker-Assisted Development and Evaluation of Near-Isogenic Lines for Broad-Spectrum Powdery Mildew Resistance Gene *Pm2b* Introgressed into Different Genetic Backgrounds of Wheat

**DOI:** 10.3389/fpls.2017.01322

**Published:** 2017-07-31

**Authors:** Hongxing Xu, Yanwei Cao, Yunfeng Xu, Pengtao Ma, Feifei Ma, Liping Song, Lihui Li, Diaoguo An

**Affiliations:** ^1^Center for Agricultural Resources Research, Institute of Genetics and Developmental Biology, Chinese Academy of Sciences Shijiazhuang, China; ^2^The College of Life Science, University of Chinese Academy of Sciences Beijing, China; ^3^The National Key Facility for Crop Gene Resources and Genetic Improvement, Institute of Crop Science, Chinese Academy of Agricultural Sciences Beijing, China

**Keywords:** *Pm2b*, near-isogenic line, *Blumeria graminis*, marker-assisted selection, wheat

## Abstract

At present, most of released wheat cultivars or breeding lines in China are susceptible to powdery mildew (*Pm*) (caused by *Blumeria graminis* f. sp. *tritici*, *Bgt*), so there is an urgent need to rapidly transfer effective and broad-spectrum *Pm* resistance genes into elite cultivars/lines. Near-isogenic lines (NILs) with short target gene region are very important in molecular breeding and map-based cloning and can be developed by combining marker-assisted selection and conventional phenotypic identification. However, no *Pm* gene NILs were reported by using this method in the previous studies. A new broad-spectrum dominant resistance gene *Pm2b*, derived from the Chinese wheat breeding line KM2939, conferred high resistance to *Pm* at both the seedling and adult stages. In this study, with the aid of forward and background selection (FS and BS) using molecular markers, the *Pm2b* gene was introgressed into three elite susceptible commercial cultivars Shimai 15, Shixin 828, and Kenong 199 through the back-crossing procedure. With the appropriate backcrossing generations, selected population sizes and marker number for BS, the homozygous resistant BC_3_F_2:3_ NILs of *Pm2b* gene in the three genetic backgrounds with the highest recipient genome composition of about 99%, confirmed by simple sequence repeat markers and 660K single nucleotide polymorphic array, were developed and evaluated for the powdery mildew resistance and agronomic traits. The different resistance and similar or improved agronomic performance between *Pm2b* NILs and their corresponding recurrent parents indicated their potential value in the marker-assisted breeding of the *Pm2b* gene. Moreover, the development of four flanked diagnostic markers (CFD81, BWM25, BWM20, and BWM21) of the *Pm2* gene can effectively assist the forward selection and accelerate the transfer and use of this resistance gene.

## Introduction

Common wheat (*Triticum aestivum* L.) is one of the most important staple food crop in China and the yield of wheat can seriously affect food safety. Of the several wheat production constraints, diseases are the most important stress, which can cause significant yield losses. Among the various wheat foliar diseases, *Pm*, caused by *B. graminis* f. sp. *tritici* (*Bgt*), is one of the most prevalent diseases occurring throughout the wheat growing regions of the world, which caused severe yield damage (Bennett, 1984; Sun et al., 2015). In China, most of the wheat cultivars released in recent years are susceptible to *Pm* and account for 83.1% of the total acreage (Li et al., 2011). In 2017, up to 8 million hectares of wheat production area are vulnerable to this disease (The Occurrence Tendency Prediction of Plant Diseases and Insect Pests in China, 2017). The use of resistant cultivars and resistance genes is the most efficient, economical and environmentally safe approach to curb this disease and reduce yield losses (Wang et al., 2015).

In wheat, two kinds of genes confers resistance against *Pm*: *mlo* genes and *Pm* resistance genes. The three *MLO* homoeologs in bread wheat (*TaMLO*-*A1*, *TaMLO*-*B1*, and *TaMLO*-*D1*) encode proteins to repress defenses against *Pm* diseases. Loss-of-function *mlo* mutant in all three *MLO* copies lead to broad-spectrum and durable resistance to *Bgt* (Wang et al., 2014). Different from *mlo* genes, most of reported *Pm* resistance genes with only one copy encode R proteins, which interacted with avirulent proteins of the pathogens to confer resistance to the diseases. At present, more than 70 formally designated *Pm* resistance genes *Pm1* – *Pm58* at 53 loci (*Pm8* is allelic to *Pm17*, *Pm18* = *Pm1c*, *Pm22* = *Pm1e*, *Pm23* = *Pm4c*, and *Pm31* = *Pm21*) and more than 20 temporarily named *Pm* genes have been reported. These genes are distributed on all chromosomes (Hao et al., 2015; Petersen et al., 2015; Zhang et al., 2016; Li et al., 2017; McIntosh et al., 2017). Most of these resistance genes are major genes conferring race-specific resistance and are easily used in disease-resistance breeding. However, relatively few *Pm* genes including *Pm2a*, *Pm4a*, *Pm6*, *Pm8*, and *Pm21* have been successfully used in breeding or in the development of resistant cultivars/lines in China (Zhang et al., 2010; Huang et al., 2012). There is an urgent need to rapidly transfer effective and broad-spectrum *Pm* resistance genes into elite released cultivars.

However, the transfer and introgression of resistance genes into released cultivars is time-consuming and inefficient when based on conventional selection strategies alone. Target resistance genes of donors may be lost due to uncertain phenotype identification. The superior agronomic traits of recurrent parents are not completely restored because of recombination and difficulties in phenotypic evaluation. Furthermore, diluted or suppressed resistance and adverse linkage drag or negative associations often occur unpredictably (Friebe et al., 1994; Zeller and Hsam, 1996; Li et al., 2017). Instead, combining marker-assisted forward and background selection (FS and BS) with conventional phenotypic selection, the target resistance gene can be precisely introgressed into the wheat cultivar background in a short time pairing with few negative association. Generally the tightly linked markers to the target gene were used to trace this gene (forward selection) and the whole genome markers were used to detect the genetic similarity between the progeny plants with their recurrent parents (BS). With the help of MAS, more and more resistance genes in wheat have been transferred into recurrent parent backgrounds (Vida et al., 2009; Kumar et al., 2010; Xue et al., 2010; Elkot et al., 2015; Guo et al., 2015; Yaniv et al., 2015).

A better way to utilize target resistance gene is to develop its NILs in the genetic backgrounds of elite cultivars and use those NILs in the molecular MAB (Tanksley et al., 1996; Zhou et al., 2005). In addition, NILs of resistance genes are valuable for their validation and determination of gene effects, differential expression profiling, fine mapping and map-based cloning (Xue et al., 2010). With the help of some closely linked markers, NILs for some resistance genes in wheat have been developed (Xue et al., 2010; Khanna et al., 2015; Habib et al., 2016; Zheng et al., 2017). However, in wheat, no NIL for *Pm* resistance gene has been developed through MAS.

Chinese wheat breeding line KM2939 confers high resistance to *Pm* at both the seedling and adult stages. It carried a broad-spectrum dominant resistance gene *Pm2b*, which was mapped on chromosome 5DS (Ma et al., 2015a). In this study, the *Pm2b* gene was introgressed into the backgrounds of commercial cultivars SM15, SX828 and KN199 with high yield and susceptibility to *Pm*, which are widely grown on the North China Plain (Ma et al., 2015a), and the NILs of *Pm2b* from different genetic backgrounds were developed and evaluated for their *Pm* resistance and agronomic traits.

## Materials and Methods

### Plant Materials

Chinese winter wheat breeding line KM2939 was characterized by high resistance to *Pm* at both the seedling and adult stages and was crossed as the *Pm2b* gene donor to three high yield and *Pm* susceptible commercial cultivars SM15 (Jimai38/92R137//Jimai 38), SX828 (422/Shixin63//612), and KN199 (Shi4185/Kenong 9204), as the recurrent parents. All the progeny plants/lines, derived from the combinations of KM2939/SM15, SX828/KM2939, and KN199/KM2939, were used to develop *Pm2b* NILs in different genetic backgrounds. The BC_2_F_1_ population of KM2939/SM15 was also used to genotype *Pm2b* gene and develop diagnostic markers (Ma et al., 2015a).

### Marker Analysis

Marker CFD81 was regarded as a co-dominant diagnostic marker for the FS of *Pm2b* (Ma et al., 2015a). Furthermore, three tightly linked SSR markers *Xbwm20*, *Xbwm21*, and *Xbwm25* of *PmPB3558*, a novel *Pm2* allele (Lu et al., 2015), were used as co-dominant diagnostic markers for tracing *Pm2b* gene. Genotyping and marker map construction were as described by Ma et al. (2015a). A total of 182 SSR markers evenly distributed on all 21 wheat chromosomes (Somers et al., 2004) were chosen to assess the genetic similarity between the progeny plants and their corresponding recurrent parents (Xue et al., 2010). The wheat 660K SNP array (designed at the Chinese Academy of Agricultural Sciences and synthesized by Affymetrix^1^) analysis also was used to determine the RGC of the *Pm2b* NIL in the genetic background of SM15. PCR amplification was performed in a Veriti^®^ thermal cycler (Applied Biosystems, Foster, CA, United States) following the procedures described by Xu et al. (2015). PCR products were separated in 8% non-denaturing polyacrylamide gels with 25:1 ratios of acrylamide and bis-acrylamide, and visualized by silver-staining as described in Santos et al. (1993).

### Phenotyping

During the transfer of *Pm2b* gene, all the progeny plants were phenotyped by *Bgt* isolate E09. To verify the resistance of *Pm2b* NILs, they were tested singly by 25 single-pustule-derived *Pm* isolates (E01, E02, E05, E06, E07, E09, E11, E13, E15, E16, E17, E18, E20, E23-1, E23-2, E26, E30-1, E30-2, E31, E49, E50, Bg01, Bg02, Bg03, and Bg04) that were avirulent to *Pm2b* gene (Ma et al., 2015a) at the seedling stage and by their composite mixture at the adult stage (Zadoks et al., 1974). Reactions of plants/lines to *Bgt* isolates and infection types (ITs) on each plant were assessed on a 0–4 scale as described by Xu et al. (2015). Plants with one of the four IT 0, 0;, 1, or 2 were regarded as resistant, and those with one of the IT 3 or 4 as susceptible. All tests and identification were repeated to assure the reliability of the data and only resistant progeny plants were retained after phenotyping.

To evaluate the agronomic performance of *Pm2b* NILs, they were planted at Luancheng Agro-ecosystem Experimental Station (37°53′15″N, 114°40′47″E) together with their respective recurrent parents and donor parent during 2015–2016 in a randomized complete block design with two replicates. Each parent/line was grown 5 row plots (20 kernels/row) with 1.5 m-long, 1.0 m-width and 0.25 m apart with two replicates. In each plot, 10 plants in the middle three internal rows were sampled to investigate the following traits: PH, SNPP, and GY determined from the mean of the ten plants; SL, TSS, SSS, KNS determined from the mean of the main spikes of the ten plants; TKW evaluated after harvest by weighing three samples of 500 kernels.

### Data Analysis

Chi-squared (χ^2^) tests for goodness-of-fit in each backcross generations were used to evaluate deviations of observed data from expected segregation ratios. The software MAPMAKER/Exp (version 3.0b) was used to determine linkage with a LOD score of 3.0 as the threshold for declaration of linkage (Lander et al., 1987). Genetic distances were estimated from the recombination values using the Kosambi mapping function (Kosambi, 1943). *T*-tests were conducted between the *Pm2b* NILs and their recurrent parents for agronomic traits.

Using the formula described by Xue et al. (2010) and Ma et al. (2015a), the RGC of progeny plants was estimated as %RGC = 100%^∗^1/2(2BB + AB)/(AA+AB+BB). AA, AB and BB represented the number of genotyped marker loci of homozygous donor, heterozygous and homozygous recipient genomes, respectively.

## Results

### The Introgression of *Pm2b* Gene

Wheat breeding line KM2939, used as the *Pm2b* gene donor, was crossed with the three wheat cultivars SM15, SX828, and KN199. Their F_1_ progeny plants were then backcrossed three times with their corresponding recurrent parents. After selfing, the BC_3_F_2:3_ lines were developed. During the transfer of *Pm2b* into the recurrent parents, the resistant donor parent KM2939, recurrent parents SM15, SX828, and KN199, and all their derived generation plants were tested against *Bgt* isolate E09, which is avirulent to *Pm2b* gene at the seedling stage and their inheritance of resistance was analyzed, and only the resistant target progeny plants after FS and BS were selected for further backcrossing or selfing (**Table 1**).

**Table 1:** The seedling resistance identification with E09 isolate and marker-assisted selection for lines of *Pm2b* gene in the genetic backgrounds of Shimai 15 (SM15), Shixin828 (SX828), and Kenong 199 (KN199).

Generations	Resistant	Susceptible	Chi-*c*^2^	*P*-value	Target plants after FS	Selected plant	Markers for BS	RGC%
KM2939 × SM15 BC_1_F_1_	36	29	*c*^2^_1:1_ = 0.55	0.46	33	KS-6	101	89.1
KM2939 × SM15 BC_2_F_1_	236	255	*c*^2^_1:1_ = 0.66	0.42	209	KS-6-88	101	96.5
KM2939 × SM15 BC_3_F_1_	21	30	*c*^2^_1:1_ = 1.25	0.26	20	KS-6-88-11	101	98.0
KM2939 × SM15 BC_3_F_2_	68	20	*c*^2^_3:1_ = 0.14	0.71	22	KS-6-88-11-2	101	99.0
KM2939 × SM15 BC_3_F_2:3_	23RR+45Rr	20rr	*c*^2^_1:2:1_ = 0.25	0.88	–	–	–	–
SX828 × KM2939 BC_1_F_1_	13	15	*c*^2^_1:1_ = 0.036	0.85	13	KSX-2	94	87.2
SX828 × KM2939 BC_2_F_1_	11	13	*c*^2^_1:1_ = 0.042	0.84	11	KSX-2-3	94	93.6
SX828 × KM2939 BC_3_F_1_	26	28	*c*^2^_1:1_ = 0.074	0.79	24	KSX-2-3-15	94	96.2
SX828 × KM2939 BC_3_F_2_	71	25	*c*^2^_3:1_ = 0.056	0.81	23	KSX-2-3-15-22	94	98.9
SX828 × KM2939 BC_3_F_2:3_	24RR+47Rr	25rr	*c*^2^_1:2:1_ = 0.063	0.97	–	–	–	–
KN199 × KM2939 BC_1_F_1_	12	10	*c*^2^_1:1_ = 0.045	0.83	12	KK-3	99	87.8
KN199 × KM2939 BC_2_F_1_	10	11	*c*^2^_1:1_ = 0.048	0.83	10	KK-3-4	99	93.9
KN199 × KM2939 BC_3_F_1_	72	70	*c*^2^_1:1_ = 0.028	0.87	69	KK-3-4-31	99	98.5
KN199 × KM2939 BC_3_F_2_	78	27	*c*^2^_3:1_ = 0.029	0.87	24	KK-3-4-31-54	99	99.0
KN199 × KM2939 BC_3_F_2:3_	25RR+53Rr	27rr	*c*^2^_1:2:1_ = 0.086	0.96	–	–	–	–

*BS, background selection; FS, forward ground selection; RGC, recipient genome composition.*

### Development of *Pm2b* Diagnostic Markers

**FIGURE 1:**
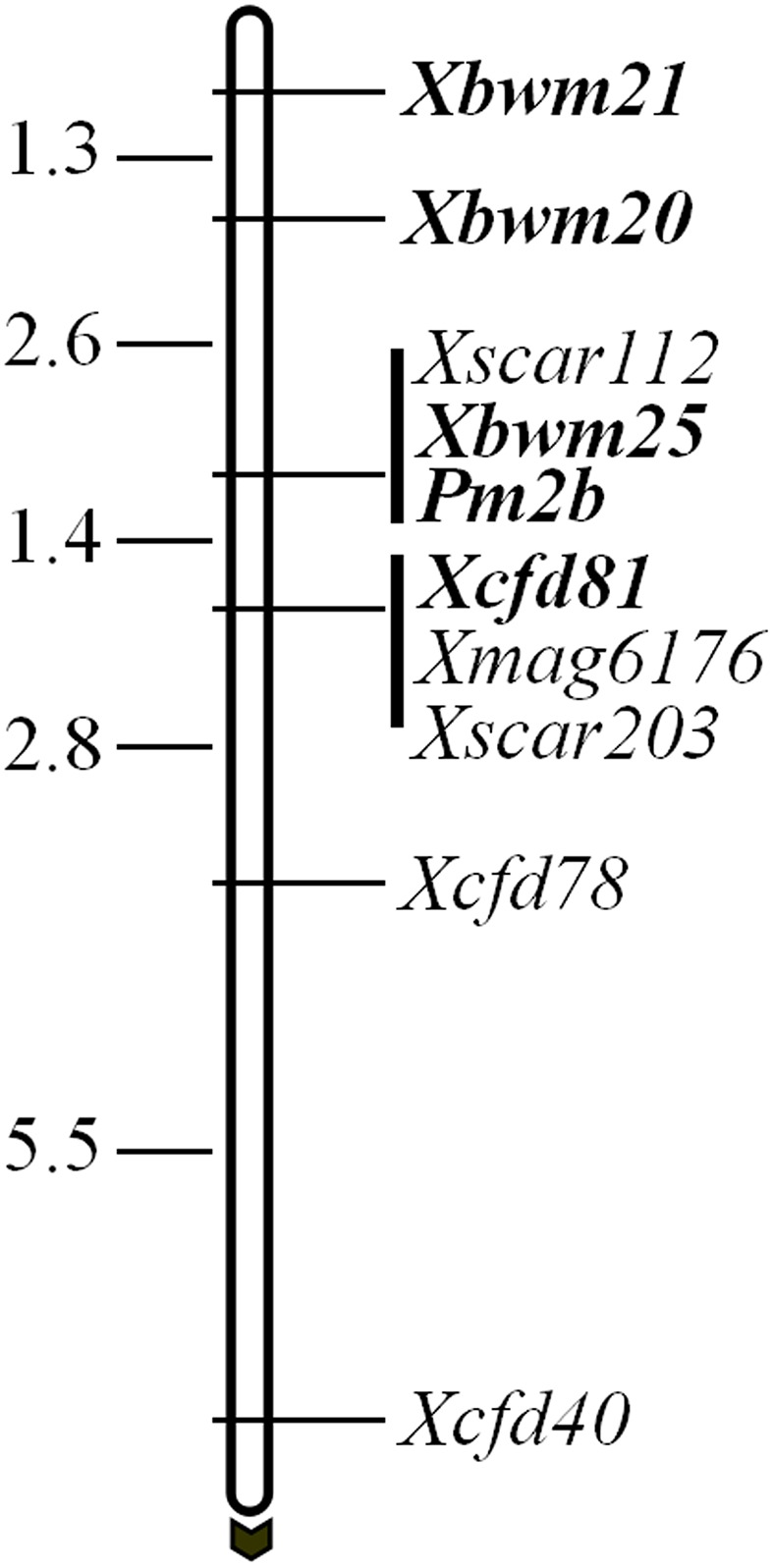
Genetic maps and diagnostic markers for *Pm2b* gene. Genetic distances are shown on the left and the black arrow indicated the centromere direction. The diagnostic markers and *Pm2b* gene are shown with bold.

In a previous study, *Pm2b* gene was reported tightly linked to four marker loci, namely *Xscar112*, *Xscar203*, *Xmag6176*, and *Xcfd81* (Ma et al., 2015a). However, only CFD81 was a co-dominant marker and could be regarded as diagnostic marker for tracing *Pm2b* gene. To identify more flanking co-dominant diagnostic markers of *Pm2b* gene, three tightly linked co-dominant SSR markers BWM20, BWM21, and BWM25 of *PmPB3558*, a novel *Pm2* allele (Lu et al., 2015), were used to map *Pm2b* gene. The results indicated that *Pm2b* gene co-segregated with *Xbwm25* and was flanked by *Xbwm20* and *Xcfd81* at genetic distances of 2.6 and 1.4 cM, respectively (**Figure 1**). In addition, *Xbwm21* was also tightly linked to *Pm2b* with a genetic distance of 3.9 cM (**Figure 1**). Thus, BWM20, BWM21, and BWM25 together with CFD81 were regarded as the diagnostic markers and were used to trace the *Pm2b* gene in this study.

### Marker-Assisted Selection for *Pm2b* Gene

During the backcross transfer of *Pm2b* gene into the genetic backgrounds of SM15, SX828 and KN199, most of the resistant progeny plants were genotyped with the four *Pm2b* diagnostic markers CFD81, BWM20, BWM21, and BWM25. As shown in the **Table 1**, all BC_1_F_1_, BC_2_F_1_, and BC_3_F_1_ progeny plants of KM2939/SM15, SX828/KM2939, and KN199/KM2939 combinations, conferring high resistance to *Bgt* isolate E09, were used in the FS by using the four *Pm2b* diagnostic markers CFD81, BWM20, BWM21, and BWM25. After FS, all the resistant backcrossed progeny plants with heterozygous genotypes for *Xcfd81*, *Xbwm20*, *Xbwm21*, and *Xbwm25*, were regarded as the target plants and chosen for assessing genetic similarity (i.e., %RGC) with their corresponding recurrent parents (**Table 1**). Then, target progeny plants with the highest %RGC were selected for further backcrossing or selfing. Finally, the homozygous resistant BC_3_F_2_ progeny plants after genotyping with the four *Pm2b* diagnostic markers CFD81, BWM20, BWM21, and BWM25, were assessed genetic similarity to their corresponding recurrent parents (**Table 1**). The homozygous resistant BC_3_F_2_ progeny plants with the highest %RGC, derived from different combinations, were regarded as the *Pm2b* NILs of their corresponding genetic backgrounds. For example, after the 36 BC_1_F_1_ resistant progeny plants of the KM2939/SM15 were genotyped with the four *Pm2b* diagnostic markers CFD81, BWM20, BWM21, and BWM25, 33 target plants were assessed for genetic similarity to SM15 (**Table 1**). After the first FS and BS, the target BC_1_F_1_ progeny plant KS-6 with the highest %RGC was further backcrossed with the recurrent parent SM15. Using continuous FS and BS, the target BC_3_F_2_ progeny plant KS-6-88-11-2 with the highest %RGC was regarded as the *Pm2b* NIL of SM15 (**Table 1**).

Based on the genetic marker map (Somers et al., 2004), a total of 182 SSR markers distributed on all 21 wheat chromosomes were chosen to assess the genetic similarity between the progeny plants and their corresponding recurrent parents. A polymorphic survey indicated that 101, 94, and 99 markers out of these 182 SSR markers were polymorphic between KM2939 and SM15, KM2939 and SX828, and KM2939 and KN199, respectively. In the BC_1_F_1_, the number of selected plants of KM2939/SM15, SX828/KM2939, and KN199/KM2939 combinations was 33, 13, and 12, and the highest %RGC were correspondingly 89.1, 87.2, and 87.8%, all much higher than 75%, which is the average expected value when no selection was used (Xue et al., 2010) (**Table 1**). In BC_2_F_1_ and BC_3_F_1_, the highest %RGC of KM2939/SM15, SX828/KM2939, and KN199/KM2939 combinations were also higher than the average expect value 87.5 and 93.75% without selection. In BC_3_F_2_, through marker-assisted FS and BS, the progeny plants KS-6-88-11-2, KSX-2-3-15-22, and KK-3-4-31-54 in the genetic backgrounds of SM15, SX828, and KN199, respectively, were selected for the homozygous *Pm2b* gene and 99% RGC (**Table 1**).

To verify the reliability of BS with genome SSR markers, the BC_3_F_2_ progeny plant KS-6-88-11-2, derived from the combination KM2939/SM15, together with its recurrent parent SM15, were genotyped with 660K wheat SNP array. Among a total of 606,470 SNPs identified, 587,587 SNPs were polymorphic between KM2939 and SM15, and 573,739, 12,488 and 1,360 were homozygous SM15/SM15, heterozygous KM2939/SM15 and homozygous KM2939/KM2939 genotypes, respectively, in KS-6-88-11-2. Thus, the %RGC of KS-6-88-11-2 was 98.7% assessed by 660K wheat SNP array, which nearly equal to the 99.0% RGC identified by genome SSR markers for BS (**Table 1**).

### Phenotype and Genotype Identification of *Pm2b* NILs

**FIGURE 2:**
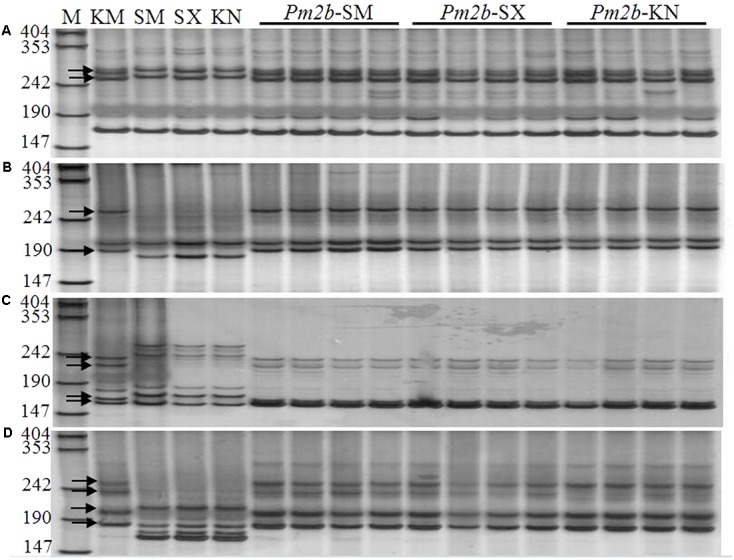
Amplification profiles generated by primers CFD81 **(A)**, BWM20 **(B)**, BWM21 **(C)**, and BWM25 **(D)** on KM2939, Shimai15, Shixin 828, Kenong 199 and their respective NILs in the backgrounds of Shimai15 (*Pm2b*-SM), Shixin 828 (*Pm2b*-SX), and Kenong 199 (*Pm2b*-KN) (Lanes 1–16). M: pUC18/*Msp*I, numbers to the left are band sizes (bp), and black arrows indicate the polymorphic bands in KM2939.

**FIGURE 3:**
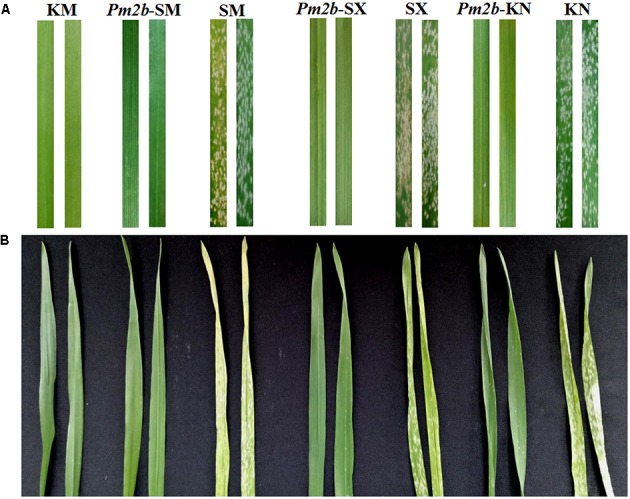
Powdery mildew reactions of KM2939, Shimai 15, Shixin 828, Kenong 199 and their respective NILs in the backgrounds of Shimai 15 (*Pm2b*-SM), Shixin 828 (*Pm2b*-SX), and Kenong 199 (*Pm2b*-KN) to *Bgt* isolate E09 at the seedling stage **(A)** and *Bgt* mixture at the adult stage **(B)**.

*Pm2b* NILs (*Pm2b*-SM, *Pm2b*-SX, and *Pm2b*-KN) in the genetic background of SM15, SX828, and KN199 were developed by selfing the BC_3_F_2_ progeny plants KS-6-88-11-2, KSX-2-3-15-22, and KK-3-4-31-54, respectively. Firstly, these *Pm2b* NILs were identified with tightly linked diagnostic markers *Xcfd81*, *Xbwm20*, *Xbwm21*, and *Xbwm25.* **Figure 2** showed the polymorphic bands about 258/274 bp (**Figure 2A**), 189/263 bp (**Figure 2B**), 222/235 and 160/167 bp (**Figure 2C**), and 236/256 and 183/202 bp (**Figure 2D**) in the *Pm2b*-SM, *Pm2b*-SX, and *Pm2b*-KN NILs amplified by *Xcfd81*, *Xbwm20*, *Xbwm21*, and *Xbwm25*, respectively. The results indicated that all *Pm2b* NILs contained the homozygous *Pm2b* gene. To verify the resistance to *Pm*, the *Pm2b*-SM, *Pm2b*-SX, and *Pm2b*-KN, together with the donor and recurrent parents, were separately inoculated with 25 *Bgt* isolates avirulent to *Pm2b* gene at the seedling stage and with mixtures of above isolates at the adult stage. All three *Pm2b* NILs resultly high resistance to *Pm* at both the seedling and adult stages (**Figure 3**).

### Evaluation of Agronomic Traits of *Pm2b* NILs

The *Pm2b* donor breeding line KM2939 had a higher PH, SL, and KNS but a lower SNPP than the three recurrent parents. Compared with the donor and the recurrent parents, the three developed *Pm2b* NILs showed improved PH and KNS and similar SSS and TKW (**Table 2**). Moreover, there was no significant difference apart from a few exceptions for SL, TSS, and GY between the *Pm2b* NILs and their respective recurrent parents. *Pm2b*-SM showed higher SL and TSS than its recurrent parent SM15, while *Pm2b*-SX had higher GY than SX828 (**Table 2**). These results indicated that the developed *Pm2b* NILs had greater KNS than their recurrent parents and PH than their donor parents. They showed the elite agronomic traits of both their donor and recurrent parents.

**Table 2:** The agronomic evaluation of near isogenic lines of *Pm2b* gene in the genetic backgrounds of Shimai 15 (SM15), Shixin828 (SX828), and Kenong 199 (KN199).

Cultivars/Lines	PH (cM)	SNPP	SL	TSS	SSS	KNS	TKW (g)	GY (g)
KM2939	91.3	6.3	10.9	20.8	0	89.0	53.9	22.9
*Pm2b*-SM	73.8^∗∗^	11.4	8.1^∗∗^	23.0^∗∗^	0.4	69.4^∗^	50.7	24.9
Shimai 15	69.3	10.0	7.2	21.3	0.8	62.5	51.7	21.8
*Pm2b*-SX	79.3^∗∗^	11.5	7.7	21.0	0.8	71.3^∗^	52.2	34.1^∗∗^
Shixin 828	64.3	10.6	7.7	21.0	0	61.0	48.8	17.5
*Pm2b*-KN	77.0^∗∗^	10.3	7.5	21.3	0.7	69.7^∗^	55.7	23.1
Kenong 199	63.0	10.8	7.2	21.2	0	60.8	51.4	22.3

*^∗^Significance between the Pm2b NILs and their respective recurrent parent at P ≤ 0.05.*

*^∗∗^Significance between the Pm2b NILs and their respective recurrent parent at P ≤ 0.01.*

*GY, grain yield per plant; KN199, Kenong 199; KNS, kernel number per spike; PH, plant height; RGC, recipient genome composition; SL, spike length; SM15, Shimai 15; SNPP, spike number per plant; SSS, sterile spikelet number per spike; SX828, Shixin 828; TKW, thousand kernel weight; TSS, total spikelet number per spike.*

## Discussion

In China, only a few *Pm* genes, which include *Pm2*, *Pm4a*, *Pm6*, *Pm8*, and *Pm21*, have been successfully used in resistant breeding in China (Zhang et al., 2010; Huang et al., 2012). Recently only *Pm2a* was still extensively used by breeders due to the lost (*Pm8*) or reduced (*Pm4a* and *Pm6*) resistance or negative linkages (*Pm21*) of the other four genes (Wang et al., 2005; Zhu et al., 2005; Ren et al., 2011). However, more and more virulent isolates against *Pm2a* have been reported in succession (Huang et al., 2012; Ma et al., 2015a; Xu et al., 2015). At the same time, at least 13 new *Pm2* alleles or closely linked genes have also been documented, such as *PmD57-5D* (Ma et al., 2011), *PmLX66* (Huang et al., 2012), *Pm48* (Gao et al., 2012), *PmX3986-2* (Ma et al., 2014), *PmW14* (Song et al., 2014), *PmPB3558* (Lu et al., 2015), *Pm2b* (Ma et al., 2015a), *PmWFJ34* (Ma et al., 2015b), *PmYB* (Ma et al., 2015c), *PmZ155* (Sun et al., 2015), *Pm2c* (Xu et al., 2015), *PmPB74* (Lu et al., 2016), *PmFG* (Ma et al., 2016). These resistance genes can make up or replace *Pm2a* for resistance breeding. However, so far few genes have been transferred into susceptible elite cultivars (Ma et al., 2015a).

Near-isogenic lines with shortest target gene region and highest %RGC are very important in molecular breeding, which can be developed by combining MAS and phenotypic selection. However, no *Pm* gene NILs have been created using this method in any previous study. In this study, the *Pm2b* gene conferring high *Pm* resistance at both the seedling and adult stages was introgressed into the susceptible elite cultivars SM15, SX828, and KN199, which were developed into the corresponding NILs by combining MAS with phenotypic evaluation. Multiple *Bgt* isolates tests for the *Pm2b* NILs demonstrated that they carried *Pm* resistance unlike the recurrent parents SM15, SX828, and KN199. These results not only validated the previous target gene mapping studies, but also further demonstrated the feasibility of MAB for *Pm* resistance gene *Pm2b*.

During the backcross transfers of *Pm2b* gene into the genetic backgrounds of the recurrent parents, it was essential to combine phenotypic identification with MAS. The strategy ensured the successful development of *Pm2b* NILs in different genetic backgrounds. All the progeny plants were phenotyped for their resistance to E09 isolate and genotyped. Then, resistant progeny plants were implemented by both marker-assisted FS and BS. To avoid the loss of target resistance gene because of double crossover, which occurs with increasing probability in successive backcrosses, multiple markers covering the target regions were used for FS. In addition, large target intervals are often accompanied by deleterious linkage drag (Friebe et al., 1994; Zeller and Hsam, 1996; Li et al., 2017), so tightly linked co-dominant markers of the *Pm2b* gene for FS minimized negative associations and ensured the precise transfer of target gene. Through both FS and BS, homozygous *Pm2b* NILs were obtained in the target regions and characterized as about 99% RGC after only three generations of backcrosses and one generation of selfing (**Table 1**). Generally two to four more generations are needed to achieve a similar goal with the conventional selection alone (Xue et al., 2010). Furthermore, the appropriate backcrossing generations, selected population sizes and marker number for BS assisted in the development of *Pm2b* NILs with the highest %RGC of 99%. During the BS procedure, selected progeny plants of every generation had a RGC significantly higher than the expected value (**Table 1**). Generally, through backcrossing three generations and selfing with a population of about 100 selected progeny plants, the NILs with the highest %RGC of 99% could be obtained with the BS for more than 100 genome SSR markers. In addition, 660K SNP array played an important role in precisely assessing the %RGC of *Pm2b* NIL compared with genome SSR markers. These results indicated that compared with conventional selection, MAS generation by generation was a significantly faster approach for recovery of the recurrent genetic backgrounds than conventional breeding.

This study compared the recurrent parents with the developed *Pm2b* NILs and showed improved agronomic traits, especially for KNS. Therefore, significantly improved resistance to *Pm* was combined with improved agronomic traits in the *Pm2b* NILs compared with their respective recurrent parents, which shows that the *Pm2b* gene can be easily used in the resistant breeding programs with the help of MAS. Moreover, these NILs will be valuable for the molecular design breeding, high-resolution mapping and forward map-based cloning of this gene.

## Conclusion

This study showed: (1) By combining MAS and phenotypic selection, a new broad-spectrum *Pm* resistance gene *Pm2b* was introgressed into the genetic backgrounds of three elite susceptible commercial cultivars and three *Pm2b* NILs with the RGC about 99% were developed; (2) the appropriate backcrossing generations (three generations), selected population sizes (about 100 progeny plants) and marker number for BS (more than 100 markers) could be used to develop the *Pm2b* NILs with the highest %RGC of 99%; (3) the %RGC of *Pm2b* NILs could be precisely assessed combining genome SSR markers and SNP array; (4) the four diagnostic markers of *Pm2b* gene can effectively accelerate the transfer and use of this resistance gene.

## Author Contributions

HX: Data analysis and manuscript preparation. YC: MAS for *Pm2b* gene. YX: Agronomic traits evaluation. PM: Mapping of *Pm2b* gene. FM: Development of *Pm2b* NILs. LS: Resistance identification. LL and DA: Experimental design and manuscript revised.

## Conflict of Interest Statement

The authors declare that the research was conducted in the absence of any commercial or financial relationships that could be construed as a potential conflict of interest.

## Footnotes

1 http://wheat.pw.usda.gov/ggpages/topics/Wheat660_SNP_array_developed_by_CAAS.pdf

## Abbreviations

*Bgt*: *Blumeria graminis* f. sp. *tritici*
BS: background selection
FS: forward ground selection
GY: grain yield per plant
ITs: infection types
KN199: Kenong 199
KNS: kernel number per spike
MAB: marker-assisted breeding
MAS: marker-assisted selection
NIL: near-isogenic line
PH: plant height
*Pm*: powdery mildew
RGC: recipient genome composition
SL: spike length
SM15: Shimai 15
SNP: single nucleotide polymorphic
SNPP: spike number per plant
SSR: simple sequence repeat
SSS: sterile spikelet number per spike
SX828: Shixin 828
TKW: thousand kernel weight
TSS: total spikelet number per spike
